# Tolerability of the SQ-standardised grass sublingual immunotherapy tablet in patients treated with concomitant allergy immunotherapy: a non-interventional observational study

**DOI:** 10.1186/s13601-016-0097-8

**Published:** 2016-03-08

**Authors:** Rainer Reiber, Martina Keller, Winfried Keller, Hendrik Wolf, Jörg Schnitker, Eike Wüstenberg

**Affiliations:** 1 HNO-Praxis, 73614 Schorndorf, Germany; 2 HNO-Gemeinschaftspraxis, 78166 Donaueschingen, Germany; 3 Clinical Development, ALK-Abelló Arzneimittel GmbH, 22763 Hamburg, Germany; 4 Institut für angewandte Statistik Dr. Jörg Schnitker GmbH, 33602 Bielefeld, Germany; 5 Medizin, ALK-Abelló Arzneimittel GmbH, 22763 Hamburg, Germany; 6 Clinic for Otolaryngology, Universitätsklinikum Carl Gustav Carus, 01307 Dresden, Germany

**Keywords:** Allergic rhinoconjunctivitis, Polysensitization, GRAZAX, Grass pollen, Sublingual immunotherapy, Subcutaneous immunotherapy, Multiple allergy immunotherapy, Tolerability

## Abstract

**Background:**

The majority of allergic patients are poly-sensitized. For causal treatment by allergy immunotherapy (AIT) a single or few allergen products containing the clinically most relevant allergens are applied, but few data on tolerability of multiple application of AIT is available. The aim of our study was to investigate safety and tolerability in patients who started treatment by sublingual immunotherapy (SLIT) with the standardised SQ^®^ grass SLIT-tablet and were treated with concomitant AIT products.

**Methods:**

In a non-interventional, open-label, observational study in Germany treatment of patients with the SQ^®^ grass SLIT-tablet and concomitant AIT (SCIT or SLIT) was documented between January 2012 and January 2014. Patients were followed at visits at first administration of the SQ^®^ grass SLIT-tablet and after 1–3 months of treatment. Tolerability of the treatment with the SQ^®^ grass SLIT-tablet and concomitant AIT were assessed by the physician and administration of AIT and adverse events (AEs) were recorded by the patients in diaries. AEs and adverse drug reactions (ADRs) were coded by using the Medical Dictionary for Regulatory Activities.

**Results:**

In total, 181 patients were documented by 48 allergists and 160 patients treated with a concomitant AIT (SCIT 130, SLIT 30). AEs were reported in 58 (36.3 %) patients with concomitant AIT, and AEs considered related with the SQ^®^ grass SLIT-tablet in 49 (30.6 %) and with concomitant AIT in 18 (11.3 %) patients. Treatment was discontinued due to ADRs in 12 (7.5 %) patients and severity of ADRs was assessed mild or moderate in 29 (18.1 %), and severe in 20 (12.5 %) patients. Most common reactions were localised at the application site of the SQ^®^ grass SLIT-tablet as oral pruritus, throat irritation, oedema mouth and paraesthesia oral; no serious ADRs were reported. Overall tolerability of the SQ^®^ grass SLIT-tablet if given with concomitant AIT was assessed as “good” or “very good” by 91.0 % of patients and 91.6 % of physicians.

**Conclusions:**

In comparison to data from previous studies no increase in frequency of AEs or change in the tolerability profile was observed when SLIT with the SQ^®^ grass SLIT-tablet was administered with concomitant SCIT or SLIT.

## Background

According to epidemiological data [1, 2], the majority of allergic patients are poly-sensitized, with their pattern of sensitization varying with the geographical region [3]. Today, the treatment of allergic diseases is based on allergen avoidance, pharmacotherapy for symptom relief, and allergy immunotherapy (AIT) [4]. For specific allergy treatment by AIT allergens are applied that have been identified as causal for allergic symptoms in order to modify the immune response to induce specific immunological tolerance [5]. The common practice of AIT, i.e. with subcutaneous application is different in Europe and the USA. While in the US patients are treated by preparations containing all allergens for which a sensitization has been detected as a mixture, in Europe AIT products containing single allergens are used and one or a few different allergen products are applied to treat the clinically most important seasonal and/or perennial allergies [3, 6].

Subcutaneous allergen injections have been primarily the main approach for the administration of AIT, however, this has subsequently been extended to sublingual administration, which offers several advantages compared with the subcutaneous route, including a better safety profile, increased convenience and at home administration [7–10].

The majority of patients treated with AIT may probably develop symptoms caused by several allergens over an extended period of the year, but only a small proportion of these patients are probably treated with more than one single allergen product. In a real-life study with the SQ^®^ grass SLIT-tablet in Germany including 1109 patients only 75 (6.8 %) were reported to be concomitantly treated with another AIT [11].

The standardised sublingual grass allergy immunotherapy tablet (SQ^®^ grass SLIT-tablet), GRAZAX^®^ (*Phleum pratense* 75,000 SQ-T/2,800 BAU, ALK-Abelló, Hørsholm, Denmark), developed for sublingual application in patients with grass pollen induced rhinoconjunctivitis and investigated in more than 5600 patients in controlled clinical trials in Europe and USA [12–25], is a European wide approved SLIT-tablet that was launched in November 2006 in Germany.

AIT has been attributed with altering the natural course of the disease, thereby entailing sustained reductions in symptoms and disease modifying effect [24]. This has been demonstrated in placebo-controlled clinical trials with the standardised SQ^®^ SCIT product (Alutard SQ^®^ *Phleum pratense*), [26] and also with the SQ^®^ grass SLIT-tablet (GRAZAX^®^), [21, 22].

The safety data obtained for the SQ^®^ grass SLIT-tablet during the clinical development program have shown that the most frequently reported adverse events (AEs) were mild to moderate, transient application-site related events with the most common reactions being oral pruritus, mouth oedema, ear pruritus, and throat irritation. In clinical practice, the question arises whether the combination of treatment with the SQ^®^ grass SLIT-tablet and a concomitant AIT product, applied as sublingual (SLIT) or subcutaneous immunotherapy (SCIT), is comparably safe and well tolerated as the mono-therapy.

The aim of the present non-interventional, observational and open-label study was, thus, to investigate the safety and tolerability of the SQ^®^ grass SLIT-tablet in patients who received a concomitant further AIT during routine treatment in several allergists’ practices in Germany.

## Methods

### Study design

This study was multi-centre, open, uncontrolled, and observational according to non-interventional post-authorization surveillance studies mentioned in the German drug law for recording of data concerning tolerability and routine application of drugs after marketing authorization. These studies are explicitly excluded from the area of application of the EU-guideline on clinical trials [4] and are, thus, not conducted according to Good Clinical Practice (GCP) guidelines [27]. All patients included were treated with the standardised SQ^®^ grass SLIT-tablet (GRAZAX^®^, ALK-Abelló, Denmark) following the specifications for administration in the Summary of product characteristics (SmPC). Data were analysed by epidemiological methods. Centers were distributed all over Germany and were asked to record data on patients in a consecutive order dependent on the patient’s willingness to participate in the study in order to avoid a selection bias.

### Allergy immunotherapy

Treatment with the oral dispersible SQ^®^ grass SLIT-tablet, GRAZAX^®^ (*Phleum pratense* 75,000 SQ-T/2,800 BAU) was initiated after the grass pollen seasons in 2012 and 2013. The first administration of the SQ^®^ grass SLIT-tablet was performed at the physician’s office at visit 1 (V1) where patients stayed for at least 30 min after the first dosing, in order to enable patient and physician to discuss any side effects. Subsequently, the SQ^®^ grass SLIT-tablet was taken by the patient at home. After V1, patients were followed at visit 2 (V2) after 1 or 3 months treatment dependent on whether 30 or 100 tablets had been prescribed at V1. Together with the data on administration of the SQ^®^ grass SLIT-tablet data on safety and tolerability of any SCIT or SLIT products available in Germany for routine treatment were planned to be recorded if these had been initiated before, at the same time or after first administration of the SQ^®^ grass SLIT-tablet during the observation period of the study.

### Ethics and data protection

According to German drug law, non-interventional post-marketing studies are notified to the authorities. The study was approved by the Ethics Committee of the Landesärztekammer Baden-Württemberg (Reference No. F-2011-075) and the consent of the patients for collection of their data was obtained. The decision of the physician to prescribe SLIT with the SQ^®^ grass SLIT-tablet and concomitant AIT has been taken independently from the inclusion of the patient in the study. For recording and evaluation of data patients were assigned a 3-digit patient number. Direct identification of the patients was restricted to the physicians’ offices that participated in the study.

### Patients

In the study 181 patients were included from 48 allergists’ offices distributed over Germany.

Patients with a diagnosis of grass pollen-induced rhinitis and/or conjunctivitis (according to symptoms and skin prick test and/or sIgE) with or without asthma with clinically relevant symptoms that were treated with the SQ grass SLIT-tablet and a concomitant AIT (SCIT or SLIT) and who had no contraindications to a prescription according to the SmPC of GRAZAX^®^ were eligible to be documented in this study.

Indications according to the SmPC are: Disease-modifying treatment of grass pollen induced rhinitis and conjunctivitis in adults and children (5 years or older) with clinically relevant symptoms and diagnosed with a positive skin prick test and/or specific IgE test to grass pollen. Contraindications are: Hypersensitivity to any of the excipients, malignancy or systemic diseases affecting the immune system e.g. autoimmune diseases, immune complex diseases or immune deficiency diseases, inflammatory conditions in the oral cavity with severe symptoms such as oral lichen planus with ulcerations or severe oral mycosis. Patients with uncontrolled or severe asthma (in adults: FEV_1_ < 70 % of predicted value after adequate pharmacologic treatment, in children: FEV_1_ < 80 % of predicted value after adequate pharmacologic treatment) should not be treated with GRAZAX^®^ immunotherapy.

### Assessments

In the present study, safety and tolerability of the SQ^®^ grass SLIT-tablet was planned to be assessed when administered with concomitant AIT.

**Fig. 1:**
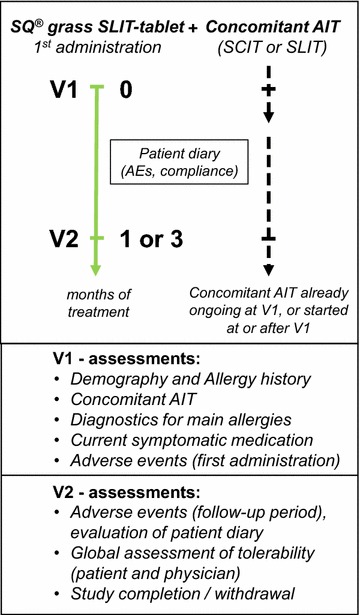
Study diagram

Evaluation of tolerability was based on AEs recorded by the physician after the first administration of the SQ^®^ grass SLIT-tablet, when giving injections of concomitant SCIT(s) and recorded by the patient in a diary during the observation period. The time schedule and the major assessments of the study are illustrated in Fig. 1.

At V1 when the patient was included in the study, demographic data and data on the allergy history including age at first appearance of symptoms, clinical manifestation of the allergy (rhinitis/conjunctivitis/asthma/atopic dermatitis/other), other allergies, the diagnostics performed for the main allergies that were treated by AIT (grass pollen and 1 or 2 concomitant allergies), any previous AIT, and concomitant treatments by AIT or other medications due to concomitant diseases were recorded. The actual anti-allergic medication used was recorded (topical or oral antihistamines/nasal or oral corticosteroids/inhaled corticosteroids/inhaled short-acting ß_2_ agonists (SABA)/inhaled long-acting ß_2_ agonists (LABA)/other, to be specified). The first administration of the SQ^®^ grass SLIT-tablet was performed in the physician’s office where an eventual anti-allergic pre-medication was recorded and any AEs that occurred while the patient was under supervision for 30 min. An AE was defined as any untoward medical occurrence in a patient and which did not necessarily have a causal relationship with treatment. AEs that were possibly related to treatment were classified as adverse drug reactions (ADRs). The application of the SQ^®^ grass SLIT-tablet during home treatment and applications of concomitant AIT(s) and side effects were recorded by the patient in a diary. Patients were asked to specify in the diary the day and clock time of applications of the SQ^®^ grass SLIT-tablet and whether the concomitant AIT product was applied on that day; if yes, patients were asked to record the clock time of application. Furthermore, patients were instructed to record the symptoms of any side effect and to assess the severity of the side effect (mild/moderate/severe) together with the time of occurrence. Furthermore, the patients were asked to record their actions they had taken due to the side effects (no action/taking a medication (which product and due to which symptoms?)/visit the doctor/discontinuation of treatment/something else (what?)). In addition, an overall assessment of tolerability (very good/good/moderate/poor) was performed by patient and physician at V2 as final visit of the study.

AEs were specified by the physician in the case report form (CRF) as diagnosis or description and assessed by intensity (mild/moderate/severe), causality (possible/unlikely), change of treatment (no change/interruption/discontinuation), treatment by medication (yes/no), outcome (recovered/recovered with sequelae/not recovered/fatal/unknown) and seriousness (yes/no). An AE was assessed as severe when the event considerably interfered with the patient’s daily activities. A serious AE (SAE) was defined as any medical occurrence or effect that was life-threatening, required hospitalization or prolongation of hospitalization, resulted in persistent or significant disability or incapacity, resulted in death, congenital abnormalities or birth defect, or any other event judged medically important. The patients came back to the physician’s office at V2 after 1–3 months for a new prescription, dependent on whether 30 or 100 SQ^®^ grass SLIT-tablets had been prescribed after first administration. At V2 the physician interviewed the patients about side effects that had occurred between V1 and V2 during home treatment, reviewed the diary and recorded all AEs in the CRF including his/her medical assessment according to the above-mentioned criteria for specification of AEs. Finally, the continuation or discontinuation of treatment and its reasons were recorded.

SAEs were further documented on a separate report form, and if applicable according to legal pharmacovigilance procedures, they were notified to the authorities.

### Statistics

Data analysis was performed solely by descriptive statistics using minimum, maximum, median, mean, range and standard deviation for continuous data as well as frequency distributions for ordinal data. No imputation was performed in case of missing data, but all available data were used to their full extent. The principal statistical software used was SAS^®^, version 9.3. No formal sample size calculation has been made for this study. The primary objective was to record data on safety and tolerability of the SQ^®^ grass SLIT-tablet when applied with concomitant AIT. To obtain a real-life picture it was aimed to engage a large number of physicians in the study who recorded data on patients that were routinely treated with the SQ^®^ grass SLIT-tablet and one or two concomitant AITs. AEs were coded according to the current version of the Medical Dictionary for Regulatory Activities (MedDRA). ADRs were displayed for patients and on the level of events including multiple occurrences by patient.

## Results

### Patients

The study was initiated in January 2012 and the last patient completed the study in January 2014. Patient characteristics and treatment with AIT are displayed in Table 1.

**Table 1:** Patient characteristics and treatment with AIT

	Concomitant AIT	No concomitant AIT	All patients treated
Patients, n	160	21	181
Age, years, mean ± SD	32.5 ± 13.9	28.2 ± 13.3	32.0 ± 13.9
Patients 5–11 years, n (%)	10 (6.3)	3 (14.3)	13 (7.2)
Patients 12–17 years, n (%)	13 (8.1)	1 (4.8)	14 (7.7)
Patients ≥18 years, n (%)	137 (85.6)	17 (81.0)	154 (85.1)
Gender, n (%)			
Male	68 (42.5)	11 (52.4)	79 (43.6)
Female	92 (57.5)	10 (47.6)	102 (56.4)
Age at first diagnosis of allergy, years ± SD	26.8 ± 14.7	23.5 ± 13.5	26.5 ± 14.5
Major manifestations, n (%)			
Rhinitis	156 (97.5)	20 (95.2)	176 (97.2)
Conjunctivitis	99 (61.9)	16 (76.2)	115 (63.5)
Asthma	68 (42.5)	4 (19.0)	72 (39.8)
Atopic dermatitis	14 (8.8)	3 (14.3)	17 (9.4)
Main allergies (multiple ratings), n (%)			
Grass pollen	160 (100.0)	21 (100.0)	181 (100.0)
Tree pollen	101 (63.1)	7 (33.3)	108 (59.7)
House dust mite	57 (35.6)	2 (9.5)	59 (32.6)
Animal hair/dander	3 (1.9)	–	3 (1.7)
Weed pollen	3 (1.9)	–	3 (1.7)
Moulds	7 (4.4)	–	7 (3.9)
Wasp venom	1 (0.6)	–	1 (0.6)
History of AIT, n (%)	18 (11.3)	7 (33.3)	25 (13.8)
Treatment with AIT, n (%)			
SQ^®^ grass SLIT-tablet	160 (100.0)	21 (100.0)	181 (100.0)
Concomitant SCIT	130 (81.3)	–	130 (71.8)
Tree pollen	74 (46.3)	–	74 (40.9)
House dust mite	40 (25.0)	–	40 (22.1)
Other allergen	6 (3.8)^a^	–	6 (3.3)^a^
Trees + mites	6 (3.8)	–	6 (3.3)
Trees + other allergen	3 (1.9)^b^	–	3 (1.7)^b^
Mites + dog	1 (0.6)	–	1 (0.6)
Concomitant SLIT	30 (18.8)	–	30 (16.6)
Tree pollen	18 (11.3)	–	18 (9.9)
House dust mite	8 (5.0)	–	8 (4.4)
Other allergen	4 (2.5)^c^	–	4 (2.2)^c^

^a^Moulds: n = 5; weed pollen: n = 1

^b^Cat: n = 1; weed pollen: n = 1; wasp venom: n = 1

^c^Cat: n = 1; moulds: n = 2; weed pollen: n = 1

First administration of the SQ^®^ grass SLIT-tablet in the physician’s office was recorded in 181 patients by 48 allergist’s offices. The study was planned to record only patients who were concomitantly treated with another AIT, in 21 patients, however, no concomitant AIT had been applied. Therefore, the data are presented separately for patients with concomitant AIT (N = 160), patients without concomitant AIT (N = 21) and total patients (N = 181). The majority of patients included in the study received predominantly one concomitant SCIT product (75.0 %) and in rare cases two (6.3 %) concomitant SCIT products, 18.8 % of patients were treated with a single concomitant SLIT product. Most patients were concomitantly treated against allergy to tree pollen (56.9 % SCIT, 60.0 % SLIT), and fewer patients against house dust mites (30.8 % SCIT, 26.7 % SLIT); few patients were concomitantly treated against allergy to moulds, weed pollen, animal dander and wasp venom (4.6 % SCIT, 13.3 % SLIT). The concomitant AIT had been started before the first administration of the SQ^®^ grass SLIT-tablet in 51.5 % of patients treated with SCIT and 50.0 % of patients treated with SLIT; it was initiated at the same day of first administration of the SQ^®^ grass SLIT-tablet in 23.1 % of patients with SCIT and in 39.3 % with SLIT, and after first administration of the SQ^®^ grass SLIT-tablet in 25.4 % with SCIT and 10.7 % with SLIT. In the group of patients with an initiation of the concomitant AIT after first administration of the SQ^®^ grass SLIT-tablet the median period of parallel administration of the SQ^®^ grass SLIT-tablet together with concomitant SCIT was 67.2 % (6.6–99.0 %) and with concomitant SLIT 60.9 % (21.3–87.9 %) of the observation period.

Discontinuations of treatment are shown in Table 2.

**Table 2:** Discontinuations of treatment in the course of the study

	Concomitant AIT n (%)	No concomitant AIT n (%)	All patients treated n (%)
*Patients*	160	21	181
Discontinuation due to			
AEs	14 (8.8)	1 (4.8)	15 (8.3)
Patient moved	1 (0.6)	1 (4.8)	2 (1.1)
Withdrawal of consent	1 (0.6)	–	1 (0.6)
Tablet application too complicated	1 (0.6)	–	1 (0.6)
Not returned after 1st administration of SQ^®^ grass SLIT-tablet	2 (1.3)	1 (4.8)	3 (1.7)
Patients continuing treatment	141 (88.1)	18 (85.7)	159 (87.8)

### Tolerability

A summary of all AEs and ADRs reported during the observation period are shown in Table 3. In 29/160 (18.1 %) patients treated with the SQ^®^ grass SLIT-tablet and concomitant AIT, severity of ADRs was assessed mild or moderate and assessed severe in 20/160 (12.5 %) patients. Treatment with the SQ^®^ grass SLIT-tablet was discontinued due to ADRs in 12/160 (7.5 %) patients treated with the SQ^®^ grass SLIT-tablet and concomitant AIT. AEs with possible relationship to the SQ^®^ grass SLIT-tablet (ADRs) reported in ≥1 % of patients are displayed as MedDRA system organ classes (SOCs) and preferred terms (PTs) in Table 4. Most frequent reactions (in ≥ 5 % of patients) were oral pruritus, throat irritation, oedema mouth and paraesthesia oral followed by ear pruritus, sneezing, pruritus and dysphagia (between 3.3 and 3.9 % of patients). No serious ADRs according to the criteria for seriousness described in the Methods section were observed. ADRs related to the concomitant AIT occurred in 17 (SCIT, 13.1 %) and 1 (SLIT, 3.3 %) patients (Table 5).

**Table 3:** Summary of patients with AEs and ADRs to treatment with the SQ^®^ grass SLIT-tablet

	Concomitant AIT n (%), E	No concomitant AIT n (%), E	All patients treated n (%), E
*Patients*	160	21	181
AEs, total	58 (36.3), 402	8 (38.1), 37	66 (36.5), 439
ADRs: SQ^®^ grass SLIT-tablet	49 (30.6), 358	8 (38.1), 37	57 (31.5), 395
Medical measures (no)	39 (24.4), 294	5 (23.8), 33	44 (24.3), 327
Medical measures (yes)	10 (6.3), 64	3 (14.3), 4	13 (7.2), 68
Severity: mild	15 (9.4), 136	5 (23.8), 34	20 (11.0), 170
Moderate	14 (8.8), 140	3 (14.3), 3	17 (9.4), 143
Severe	20 (12.5), 80	–	20 (11.0), 80
Missing value	–, 2	–	–, 2
SQ^®^ grass SLIT-tablet discontinued	12 (7.5), 25	1 (4.8), 2	13 (7.2), 27
ADRs: concomitant AIT	18 (11.3), 99	–	18 (9.9), 99

**Table 4:** ADRs related to the SQ^®^ grass SLIT-tablet in ≥1 % of patients

MedDRA system organ class MedDRA preferred term	Concomitant AIT n (%), E	No concomitant AIT n (%), E	All patients treated n (%), E
*Patients*	160	21	181
*All patients with ADRs*	49 (30.6), 358	8 (38.1), 37	57 (31.5), 395
Ear and labyrinth disorders	8 (5.0), 18	–	8 (4.4), 18
Ear pruritus	7 (4.4), 17	–	7 (3.9), 17
Eye disorders	6 (3.8), 11	–	6 (3.3), 11
Eye pruritus	3 (1.9), 4	–	3 (1.7), 4
Eye irritation	2 (1.3), 6	–	2 (1.1), 6
Gastrointestinal disorders	35 (21.9), 131	5 (23.8), 29	40 (22.1), 160
Oral pruritus	14 (8.8), 34	1 (4.8), 6	15 (8.3), 40
Oedema mouth	10 (6.3), 16	3 (14.3), 3	13 (7.2), 19
Paraesthesia oral	8 (5.0), 21	3 (14.3), 10	11 (6.1), 31
Dysphagia	6 (3.8), 11	–	6 (3.3), 11
Hypoaesthesia oral	4 (2.5), 5	1 (4.8), 2	5 (2.8), 7
Oral discomfort	4 (2.5), 6	–	4 (2.2), 6
Lip swelling	2 (1.3), 2	2 (9.5), 6	4 (2.2), 8
Tongue pruritus	3 (1.9), 4	–	3 (1.7), 4
Nausea	3 (1.9), 4	–	3 (1.7), 4
Swollen tongue	2 (1.3), 2	–	2 (1.1), 2
Lip pruritus	2 (1.3), 2	–	2 (1.1), 2
Glossodynia	2 (1.3), 4	–	2 (1.1), 4
Abdominal pain upper	2 (1.3), 2	–	2 (1.1), 2
General disorders and administration site conditions	11 (6.9), 38	–	11 (6.1), 38
Fatigue	3 (1.9), 13	_	3 (1.7), 13
Sensation of foreign body	3 (1.9), 8	–	3 (1.7), 8
Injection site pruritus	2 (1.3), 2	–	2 (1.1), 2
Infections and infestations	2 (1.3), 6	–	2 (1.1), 6
Nasopharyngitis	2 (1.3), 6	–	2 (1.1), 6
Nervous system disorders	6 (3.8), 26	2 (9.5), 3	8 (4.4), 29
Headache	3 (1.9), 19	2 (9.5), 3	5 (2.8), 22
Dizziness	2 (1.3), 3	–	2 (1.1), 3
Respiratory, thoracic and mediastinal disorders	30 (18.8), 94	3 (14.3), 4	33 (18.2), 98
Throat irritation	13 (8.1), 34	–	13 (7.2), 34
Sneezing	7 (4.4), 11	–	7 (3.9), 11
Cough	5 (3.1), 8	–	5 (2.8), 8
Oropharyngeal pain	3 (1.9), 8	–	3 (1.7), 8
Pharyngeal oedema	2 (1.3), 3	1 (4.8), 2	3 (1.7), 5
Nasal discomfort	2 (1.3), 2	1 (4.8), 1	3 (1.7), 3
Rhinorrhea	3 (1.9), 4	–	3 (1.7), 4
Dyspnoea	2 (1.3), 5	–	2 (1.1), 5
Nasal congestion	2 (1.3), 5	–	2 (1.1), 5
Rhinitis allergic	2 (1.3), 2	–	2 (1.1), 2
Dry throat	2 (1.3), 2	–	2 (1.1), 2
Skin and subcutaneous disorders	9 (5.6), 23	1 (4.8), 1	10 (5.5), 24
Pruritus	6 (3.8), 17	–	6 (3.3), 17
Rash	3 (1.9), 3	–	3 (1.7), 3

**Table 5:** ADRs related to the concomitant AIT in ≥1 % of patients (in at least one treatment group)

MedDRA system organ class MedDRA preferred term	Concomitant SCIT n (%), E	Concomitant SLIT n (%), E	Concomitant AIT n (%), E
*Patients*	130	30	160
*All patients with ADRs*	17 (13.1), 96	1 (3.3), 3	18 (11.3), 99
Ear and labyrinth disorders	3 (2.3), 5	–	3 (1.9), 5
Ear pruritus	2 (1.5), 3	–	2 (1.3), 3
Gastrointestinal disorders	4 (3.1), 10	1 (3.3), 1	5 (3.1), 11
Abdominal pain	–	1 (3.3), 1	1 (0.6), 1
General disorders and administration site conditions	13 (10.0), 39	–	13 (8.1), 39
Fatigue	3 (2.3), 12	–	3 (1.9), 12
Injection site pruritus	4 (3.1), 7	–	4 (2.5), 7
Injection site swelling	5 (3.8), 5	–	5 (3.1), 5
Respiratory, thoracic and mediastinal disorders	6 (4.6), 20	1 (3.3), 1	7 (4.4), 21
Oropharyngeal pain	–	1 (3.3), 1	1 (0.6), 1
Sneezing	3 (2.3), 4	–	3 (1.9), 4
Skin and subcutaneous tissue disorders	4 (3.1), 9	1 (3.3), 1	5 (3.1), 10
Pruritus	2 (1.5), 7	–	2 (1.3), 7
Rash	1 (0.8), 1	1 (3.3), 1	2 (1.3), 2

### Compliance

Patient diaries could be evaluated in 126 patients of the total 181 patients (69.6 %) and in 114 patients who had received a concomitant AIT (71.3 %). According to the diary records, 89 (70.6 %) patients of all patients with a diary were compliant by ≥80 % with the daily intake of the SQ^®^ grass SLIT-tablet, 25 (19.8 %) by 50–79 % and 12 (9.5 %) by <50 %.

## Discussion

The SQ^®^ grass SLIT-tablet has been launched 2006 in Germany according to a European wide approval for marketing based on randomised controlled trials demonstrating efficacy, safety and tolerability. In the present study, safety and tolerability of the SQ^®^ grass SLIT-tablet administered with concomitant AIT (81 % SCIT and 19 % SLIT) were assessed in 160 patients in a routine clinical setting. AEs considered related with the SQ^®^ grass SLIT-tablet were reported in 49 (30.6 %) patients and in 18 (11.3 %) patients with the concomitant AIT, discontinuations due to AIT related AEs (ADRs) were recorded in 12 (7.5 %) patients; severity of ADRs was assessed in 29 (18.1 %) mild to moderate and severe in 20 (12.5 %) patients. Overall tolerability was assessed “good” or “very good” by 91.0 % of patients and 91.6 % of physicians.

Safety data from two studies on a multiple treatment in poly-sensitized patients have been discussed in review articles by Passalaqua [3] and Calderon [6] published recently. In a post marketing survey, treatment with SLIT was performed with single allergens or a mixture of allergens in 433 children, out of which 254 received the mixture of several allergens. No increased rates of side effects have been observed in the children that received multiple allergens [28].

In a retrospective analysis on 147 poly-sensitized patients treated with SCIT by one single or two parallel injections of single allergen extracts (various pollen, house dust mite, moulds, animal dander and Hymenoptera venom) a slightly higher but not significant rate of ADRs was observed during the dose-increase phase in the parallel injection group [6, 29]. To our knowledge, no further systematically recorded data on the safety and tolerability of SLIT (i.e. SLIT-tablet) concomitantly administered with other AIT (SCIT or SLIT) have been published.

In a previous non-interventional study with the SQ^®^ grass SLIT-tablet performed in Germany only a minor number of poly-sensitized patients with multiple clinical relevant allergies was treated with more than one AIT product [11]. The number of patients treated with the SQ^®^ grass SLIT-tablet and concomitant AIT available for collecting data on safety and tolerability within a real-life setting appears, thus, to be limited. Our non-interventional, observational study was designed to observe the first 1–3 months of treatment with the SQ^®^ grass SLIT-tablet because the highest frequency of adverse reactions is known to occur at first administration and to decline with longer duration of treatment due to the increasing induction of tolerance to grass allergens by an effective treatment [12–25, 30]. The majority of patients who started treatment with the SQ^®^ grass SLIT-tablet included in our study were treated concomitantly with one further pollen allergen product, i.e. tree pollen allergens (60 %), and fewer patients with one perennial allergen product, i.e. house dust mite allergens (30 %). Reflecting the general use of AIT in Germany, most patients were treated with one concomitant AIT product, predominantly SCIT (75 %) and fewer patients (18.8 %) with a single concomitant SLIT product and only a small number with two concomitant AIT products, all SCIT (6.3 %). A small subgroup of patients (11.6 %) in our study did not receive a concomitant AIT. Data on this group is presented separately, but the number of patients is too small to be used for comparison of the incidence of ADRs for patients with and without concomitant AIT. Therefore, we used data from previous non-interventional studies with the SQ^®^ grass SLIT-tablet in the real-life setting for comparison of the incidence of ADRs in the group of patients with concomitant AIT of our study [11, 31, 32]. The observed incidence of ADRs of 31.5 % of 181 total patients in our study and of 30.6 % in 160 patients with concomitant AIT was at a similar level as in two previous studies with the SQ^®^ grass SLIT-tablet with a higher number of patients, one with 1109 adult patients and 27.0 % of patients with ADRs [11] and one with 1761 patients (964 adults and 797 children and adolescents) and 31.8 % of patients with ADRs [31], but lower than in another study with intra-seasonal initiation of the SQ^®^ grass SLIT-tablet in 662 adult patients in which ADRs were observed in 49.8 % of patients [32]. ADRs in our study were of mild to moderate severity in the majority of patients (20.4 %) and in fewer patients severe (11.0 %). The rate of discontinuations due to ADRs (7.5 %) in patients who received a concomitant AIT was similar as in the previous non-interventional studies [11, 31, 32].

The tolerability profile observed in our study was comparable to the one observed in the previous non-interventional real-life studies and controlled clinical trials with the SQ^®^ grass SLIT-tablet [11–25, 31, 32]. Most frequent reactions observed in our study were local reactions at the application site of the SQ^®^ grass SLIT-tablet in the mouth as oral pruritus, throat irritation, oedema mouth and paraesthesia oral (≥5 % of patients). Clearly typical side effects of SCIT were observed in the group of patients with concomitant AIT, but no interaction with side effects typical for the SQ^®^ grass SLIT-tablet was detected. The rate of reactions considered by the physicians related to the concomitant AIT was low, overall (11.3 % of the 160 patients with a concomitant AIT).

Limitations of our study are those of a prospective, open-label, un-controlled observational study in routine treatment. In order to minimize a potential investigator bias sites distributed all over Germany were involved. For reduction of a potential selection bias physicians were asked to include patients in a consecutive order according to the consent of the patients to be included in the study. The number of 160 patients with concomitant AIT and safety and tolerability data allows detecting ADRs of incidence 2 % with a probability of 95 % at least once. The interpretation of the safety and tolerability data from our study is, therefore, limited to a comparison of data for frequencies of ADRs with data from previous studies of similar design (including less than 10 % of patients with concomitant AIT) and to a comparison of the safety and tolerability profile for ADRs known to be most frequent for the application of the SQ^®^ grass SLIT-tablet during the first 1–3 months of treatment. This study period is short with respect to the evaluation of AEs by a concomitant SCIT treatment.

## Conclusions

In conclusion no increase in frequency of AEs compared with data from previous studies or change in the tolerability profile was observed when the SQ^®^ grass SLIT-tablet was administered with concomitant SCIT or SLIT in real life.

## Abbreviations

AIT: allergy immunotherapy
AE: adverse event
ADR: adverse drug reaction
FEV1: forced expiratory volume in 1 s
GCP: good clinical practice
MedDRA: medical dictionary of drug regulatory activities
PT: preferred term
SCIT: subcutaneous immunotherapy
SLIT: sublingual immunotherapy
SmPC: summary of product characteristics
SOC: system organ class
SQ: standardised quality
